# Adherence to the World Cancer Research Fund/American Institute for Cancer Research recommendations and endometrial cancer risk: a multicentric case–control study

**DOI:** 10.1017/S0007114522002872

**Published:** 2023-06-28

**Authors:** Giovanna Esposito, Federica Turati, Diego Serraino, Anna Crispo, Eva Negri, Fabio Parazzini, Carlo La Vecchia

**Affiliations:** 1 Department of Clinical Sciences and Community Health, University of Milan, Milan, Italy; 2 Unit of Cancer Epidemiology, CRO Aviano National Cancer Institute, IRCCS, Aviano, Italy; 3 Epidemiology and Biostatistics Unit, Istituto Nazionale dei Tumori – IRCCS ‘Fondazione G. Pascale’, Naples, Italy; 4 Department of Medical and Surgical Sciences, University of Bologna, Bologna, Italy

**Keywords:** Endometrial cancer, Case–control study, World Cancer Research Fund, Diet, Lifestyle, Prevention

## Abstract

The World Cancer Research Fund/American Institute for Cancer Research (WCRF/AICR) published evidence-based recommendations for cancer prevention focusing on body weight, physical activity, and diet. Our aim is to evaluate whether adherence to the WCRF/AICR recommendations could reduce endometrial cancer risk. We used data from a multicentric, Italian hospital-based case–control study (1992–2006) including 454 endometrial cancer cases and 908 age-matched controls. Adherence to the WCRF/AICR recommendations was measured using a score (range: 0–7) based on seven components: body mass index (BMI), physical activity and five dietary items; higher scores indicated higher adherence. Odds ratios (OR) were estimated by multiple (adjusted) conditional logistic regression models including terms for major confounders and energy intake. Adherence to the WCRF/AICR recommendations was inversely related to endometrial cancer risk (OR = 0·42, 95 % confidence interval (CI) 0·30, 0·61 for the highest compared with the lowest score quartile), with a significant trend of decreasing risk with increasing adherence. An inverse association was also observed for a score including only dietary recommendations (OR = 0·67, 95 % CI 0·46, 0·96 for the highest compared with the lowest score tertile). In stratified analyses, the association was stronger among women with a normal weight, those who were older, and consequently those in post-menopause, and those with ≥ 2 children. In conclusion, high adherence to the WCRF/AICR recommendations has a favourable role in endometrial cancer risk, which is not fully explained by body weight.

Endometrial cancer represents the 6th most commonly diagnosed malignancy among women, with over 417 000 new cases and 97 000 deaths in 2020^(1)^.

Endometrial cancer primarily affects post-menopausal women^(2)^. The use of oestrogenic hormone replacement therapy (HRT)^(3)^, obesity^(4–6)^ and physical inactivity^(5,7,8)^ represent the main modifiable risk factors for the disease. With the ageing of the population and the rising prevalence of obesity and sedentary lifestyle, the burden of endometrial cancer is expected to increase globally^(9)^; primary prevention of this neoplasm is therefore of paramount importance.

Dietary habits may influence endometrial cancer. High glycaemic load diet^(10,11)^ and high consumption of red and processed meat^(12–14)^ have been associated with the disease, while high consumption of coffee^(15–17)^, fibres^(18,19)^, fruit^(20)^ and vegetables^(20–22)^ may reduce the risk. However, evidence on dietary factors is still controversial^(23)^.

In 2007, the World Cancer Research Fund/American Institute for Cancer Research (WCRF/AICR) published the following evidence-based recommendations aimed at cancer prevention: (1) be as lean as possible within the normal range of body weight, (2) be physically active as part of everyday life, (3) limit consumption of energy-dense foods, (4) eat mostly foods of plant origin, (5) limit intake of red meat and avoid processed meat, (6) limit consumption of alcoholic drinks, (7) limit consumption of salt and avoid mouldy cereals or pulses, (8) avoid dietary supplements for cancer prevention, and (9) breastfeed^(24)^. In 2018, recommendations were updated with minor changes, including the avoidance of any alcohol and the avoidance of sugar-sweetened drinks as a separate recommendation^(25)^. The 2007 recommendations on limiting salt consumption and avoiding mouldy cereals or pulses were removed in the 2018 version, as these are specific for selected populations.

In several cohort, case–control and cross-sectional studies, adherence to the WCRF/AICR recommendations was associated with reduced total and cardiovascular mortality^(26)^, and reduced risks of overall^(26–28)^ and selected cancers, including those of the breast^(29–32)^, colorectum^(33–39)^, pancreas^(40,41)^, prostate^(42)^, and upper aerodigestive tract^(43)^. However, to our knowledge, no previous investigation has analysed the association of adherence to these recommendations with the occurrence of endometrial cancer.

In the current study, we evaluated whether adherence to the WCRF/AICR cancer prevention recommendations may affect endometrial cancer risk using data from a multicentric case–control study conducted in Italy.

## Materials and methods

### Study population and data collection

We analysed data from a hospital-based case–control study on endometrial cancer conducted between 1992 and 2006 in three Italian areas, that is, the greater Milan area, the provinces of Udine and Pordenone in northern Italy and the urban area of Naples in southern Italy^(44,45)^. Cases were 454 women (median age 60 years, range 18–79) diagnosed with incident histologically confirmed endometrial cancer according to the International Classification of Diseases (ICD-9-CM, code 182·0), admitted to major teaching and general hospitals of the study areas. Women diagnosed with endometrial cancer up to a year earlier and with no prior diagnosis of cancer were eligible. Controls were 908 women (median age 61 years, range 19–79) admitted to the same hospital network as cases for acute, non-neoplastic conditions, unrelated to long-term dietary modifications, that is, traumas (36 %), orthopaedic disorders (32 %), acute surgical conditions (9 %) and miscellaneous illnesses including eye, nose, ear, or skin disorders (23 %). Women with a history of hysterectomy or admitted for gynaecological or hormone-related conditions were excluded from the control group. Cases and controls were frequency matched by 5-year age group and study centre; we used a case to control ratio of 1:2 to increase the statistical power of the study. Comprising over 450 cases and 900 controls, our study has ∼90 % power to detect as statistically significant (at *α* = 0·05) an odds ratio (OR) equal or greater than 1·5 for an exposure with a prevalence of 25 % in controls. Matching was achieved by sampling as controls twice the number of cases in each 5-years age group. This was done by periodically checking the age distribution of cases within each participating centre. More than 95 % of eligible cases and a similar proportion of controls agreed to participate in the study and completed the questionnaire. This study was conducted according to the guidelines laid down in the Declaration of Helsinki and was approved by the Board of Ethics of each participating centre. Informed consent was obtained from all enrolled women.

Patients were interviewed by centrally trained personnel during their hospital stay using a standard structured questionnaire collecting information on socio-demographic characteristics and anthropometric measures (including self-reported weight before diagnosis/hospital admission), selected lifestyle habits (i.e., tobacco smoking, alcohol drinking, and physical activity), personal medical history of selected diseases, family history of cancer in first-degree relatives, menstrual and reproductive factors, and use of oral contraceptive and HRT. Body mass index (BMI) was calculated as weight divided by height^2^ (kg/m^2^). Occupational and leisure time physical activities at ages 12, 15–19, 30–39, and 50–59 were self-reported. Occupational physical activity was classified, based on the type of job, as sedentary (e.g., office worker, student), standing (e.g., shop assistant, teacher, laboratory worker), intermediate (e.g., waiter, cook, kindergarten teacher, housewife doing housework), heavy (e.g. farmer, heavy industry worker), and very heavy (e.g., construction bricklayer, athlete). As for leisure time physical activity, we asked subjects to report their usual number of hours of physical activities (including sport, cycling, etc.) per week (i.e., > 7, 5–7, 2–4, and < 2).

Information regarding the usual diet in the 2 years before cancer diagnosis (for cases) or hospital admission (for controls) was retrieved using a reproducible^(46,47)^ and valid^(48)^ food frequency questionnaire (FFQ) including seventy-eight food items or food groups and, for about half of them, their usual portion size. Subjects were asked to indicate their average weekly consumption of each item in the past 2 years. Intake of non-alcohol energy and selected nutrients was determined using an Italian food composition database^(49)^.

### World Cancer Research Fund/American Institute for Cancer Research score

We calculated a score measuring adherence to the 2018 version of the WCRF/AICR recommendations according to standard criteria proposed by Shams-White *et al.*^(50,51)^. We included seven of eight recommendations, that is, (1) be at a healthy weight, (2) be physically active, (3) eat a diet rich in vegetables, fruits and wholegrains, (4) limit consumption of *fast foods* and other processed foods high in fat, starches or sugars, (5) limit consumption of red and processed meat, (6) limit consumption of sugar sweetened, and (7) avoid consumption of alcohol. The recommendation number 3 was split into two sub-recommendations (as also suggested by the standard scoring system^(50,51)^): one on vegetables and fruits (3a) and one on wholegrains (3b). The optional recommendation on breast feeding was not included. For each recommendation, participants were assigned 1 point for complete adherence, 0·5 for partial adherence, and 0 for non-adherence. For the two sub-recommendations (i.e., 3a and 3b), participants were assigned 0·5 points for complete adherence, 0·25 for partial adherence, and 0 for non-adherence; points on the two sub-recommendations were, then, summed up. Complete, partial, and non-adherence to the recommendations were defined, respectively, as follows: (1) BMI: 18·5–24·9, 25–29·9, < 18·5, or ≥ 30 kg/m^2^ (data on waist circumference were not considered since the information was available only for a subset of women); (2) physical activity: very heavy/heavy job or ≥ 5 h/week of leisure time physical activity, medium job and ≤ 4 h/week of leisure time physical activity or standing/sedentary job and 2–4 h/week of leisure time physical activity, sedentary job and < 2 h/week of leisure time physical activity; (3a) consumption of vegetables and fruits: ≥ 400, 200–< 400, < 200 g/diet; (3b) consumption of wholegrains: ≥ 30, 15–< 30, < 15 g/diet; (4) consumption of energy-dense foods (as a proxy for the consumption of *fast foods* and other processed foods high in fat, starches or sugars): ≤ 523·0, 523·0–< 732·2, ≥ 732·2kJ/100 g/diet; (5) consumption of red and processed meat: red and processed meat < 500 and processed meat < 21 g/week, red and processed meat < 500 and processed meat 21–< 100 g/week, red and processed meat ≥ 500 or processed meat ≥ 100 g/week; (6) consumption of sugar-sweetened drinks: 0, > 0–≤ 250, > 250 g/week and (7) consumption of alcohol: 0, > 0–≤ 7, > 7 drinks/week (see details in online Supplementary Table S1). The overall WCRF/AICR score was obtained as the sum of the points assigned to each recommendation; its theoretical range is from 0 to 7, with higher values indicating greater adherence to the WCRF/AICR recommendations. We also derived a dietary WCRF/AICR score summing up only the five recommendations regarding dietary habits (i.e., eat a diet rich in vegetables, fruits and wholegrains; limit consumption of *fast foods* and other processed foods high in fat, starches or sugars; limit consumption of red and processed meat; limit consumption of sugar-sweetened beverages; and avoid consumption of alcohol); its theoretical range is from 0 to 5.

### Statistical analysis

We derived the OR of endometrial cancer and the corresponding 95 % confidence intervals (CI) according to each WCRF/AICR recommendation (in three categories for complete, partial, and non-adherence), to the overall WCRF/AICR score (in approximate quartiles calculated among controls, i.e., < 3·25, 3·25–3·99, 4·00–4·49, ≥ 4·50, as well as for one-point increment) and to the dietary WCRF/AICR score (in approximate tertiles among controls: < 2·25, 2·25–2·99, ≥ 3·00, as well as for one-point increment). We used multiple (adjusted) logistic regression models, conditioned on 5-year age group and centre, and including terms for years of education, year of interview, smoking, history of diabetes, total energy intake, age at menarche, parity, menopausal status, use of oral contraceptive and HRT. When assessing the association of single recommendations and the dietary WCRF/AICR score, we included in the model as adjustment factors terms for BMI (in categories: < 21·00, 21·00–25·99, 26·00–29·99, ≥ 30 kg/m^2^, except for the analysis on the recommendation on body fatness) and occupational and leisure time physical activity (in categories defined as the recommendation on physical activity included in WCRF score, except for the analysis on the corresponding recommendation). A few missing data on adjustment factors were replaced by the median value (continuous variables) or mode category (categorical variables) according to case/control status. In sensitivity analyses, we excluded alternately each recommendation at a time from the overall WCRF/AICR score in order to evaluate the relative impact of the single recommendations included in the score, and we re-ran the main analysis with a complete case approach.

Additionally, we estimated the OR for one-point increment in the WCRF/AICR score across strata of age, BMI, menopausal status, parity, oral contraceptive and HRT use. Heterogeneity across strata was tested by a likelihood ratio test comparing the models with and without the interaction term between the subgroup factor and the WCRF/AICR score variable. For the likelihood ratio test, we considered as significant a *P*-value < 0·10.

All the analyses were conducted using SAS software version 9.4 (SAS Institute, Inc.).

## Results

Table 1 shows the distribution of selected characteristics of endometrial cancer cases and controls. By design, cases and controls had a similar age and were hospitalised in the same centres. Compared with controls, cases had a higher BMI, reported more frequently a history of diabetes and had lower parity; they also tended to report more frequently HRT use and less frequently oral contraceptive use. No differences emerged according to the other factors considered. In our database, the WCRF/AICR score ranged from 0·5 to 6·5.

**Table 1.** Distribution of endometrial cancer cases and controls according to selected covariates, Italy, 1992–2006 (Numbers and percentages; mean values and standard deviations)

	Cases (*n* 454)		Controls (*n* 908)		*P*
	*n*	%	*n*	%	
Centre					
Milan	140	30·8	280	30·8	Matching variable
Naples	77	17·0	154	17·0	
Pordenone	237	52·2	474	52·2	
Age					
< 50	67	14·8	134	14·8	Matching variable
50–54	59	13·0	118	13·0	
55–59	81	17·8	162	17·8	
60–64	84	18·5	167	18·4	
65–69	82	18·1	165	18·2	
≥ 70	81	17·8	162	17·8	
Education (years)					
< 7	263	57·9	553	60·9	
7–11	119	26·2	225	24·8	
≥ 12	72	15·9	130	14·3	0·555
BMI (kg/m^2^)*					
< 25	126	27·8	413	45·7	
25–29·9	160	35·2	351	38·8	
≥ 30	168	37·0	140	15·5	<0·001
History of diabetes					
No	401	88·3	854	94·1	
Yes	53	11·7	54	6·0	<0·001
Parity					
0	68	15·0	126	13·9	
1	92	20·3	150	16·5	
2	169	37·2	315	34·7	
> 2	125	27·5	317	34·9	0·041
Menopausal status†					
Pre/Peri	83	18·7	174	19·3	
Post	360	81·3	726	80·7	0·794
Oral contraceptive use					
Never	408	89·9	790	87·0	
Ever	46	10·1	118	13·0	0·126
Hormone replacement therapy					
Never	405	89·2	830	91·4	
Ever	49	10·8	78	8·6	0·188
WCRF score					
Mean	3·65		3·93		< 0·001
sd	0·87		0·96		

WCRF, World Cancer Research Fund.

* 4 (0·4 %) missing values among controls.

† 11 (2·4 %) missing values among cases and 8 (0·8 %) among controls.

Table 2 provides the OR of endometrial cancer for each recommendation included in WCRF/AICR score. Complete adherence (i.e., 1 point) to the recommendation on body fatness reduced the risk of endometrial cancer by 72 % (OR = 0·28, 95 % CI 0·20, 0·39 *v*. non-adherence, *P*-value for trend < 0·001). There was an inverse association with adherence to the recommendation on red and processed meat (OR for complete *v*. non-adherence = 0·50, 95 % CI 0·24, 1·03, *P* for trend = 0·013). No significant association was found for adherence to the other recommendations.

**Table 2.** Asssociation between adherence to each recommendation included in the World Cancer Research Fund/American Institute for Cancer Research (WCRF/AICR) score and endometrial cancer risk, Italy, 1992–2006 (Odds ratios and 95 % confidence intervals; numbers and percentages)

	Cases *n*	%	Controls *n*	%	OR*	95 % CI
Recommendations						
Be at a healthy weight†						
0	172	37·9	158	17·4	1·00‡	
0·5	155	34·1	344	38·1	0·39	0·29, 0·53
1	127	28·0	402	44·5	0·28	0·20, 0·39
*P*_trend_					< 0·001	
Be physically active†						
0	42	9·3	64	7·1	1·00‡	
0·5	217	47·9	503	55·8	0·68	0·43, 1·07
1	194	42·8	335	37·1	0·94	0·57, 1·54
*P*_trend_					0·325	
Eat a diet rich in whole grains, vegetables, fruit and beans						
0	63	13·9	127	14·0	1·00‡	
0·5	329	72·5	666	73·3	0·81	0·56, 1·19
1	62	13·7	115	12·7	0·69	0·40, 1·19
*P*_trend_					0·180	
Limit consumption of energy-dense food						
0	71	15·6	129	14·2	1·00‡	
0·5	280	61·7	529	58·3	0·91	0·64, 1·30
1	103	22·7	250	27·5	0·78	0·52, 1·18
*P*_trend_					0·219	
Limit consumption of red and processed meat						
0	357	78·6	649	71·5	1·00‡	
0·5	86	18·9	214	23·6	0·75	0·55, 1·02
1	11	2·4	45	5·0	0·50	0·24, 1·03
*P*_trend_					0·013	
Limit consumption of sugar-sweetened beverages†						
0	27	5·9	50	5·5	1·00‡	
0·5	176	38·8	332	36·7	1·15	0·67, 1·98
1	251	55·3	523	57·8	1·05	0·62, 1·79
*P*_trend_					0·727	
Avoid consumption of alcohol†						
0	156	34·5	294	32·6	1·00‡	
0·5	159	35·2	337	37·4	0·87	0·65, 1·18
1	137	30·3	271	30·0	0·90	0·64, 1·25
*P*_trend_					0·505	

* Estimated from logistic regression models conditioned on age and centre and including terms for year of interview, education, BMI, physical activity, smoking, total energy intake, history of diabetes, age at menarche, menopausal status, parity, use of oral contraceptives and hormone replacement therapy, unless the variable was part of the recommendation under evaluation.

† The sum does not add up to the total because of missing data.

‡ Reference category.

**Fig. 1.**
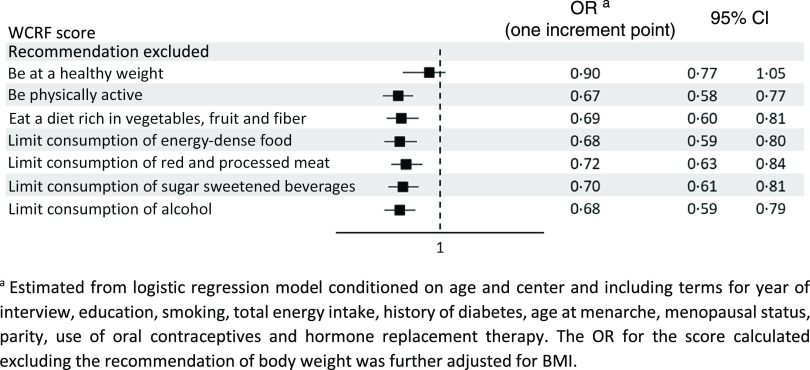
Odds ratios (OR) and corresponding 95 % confidence interval (CI) of endometrial cancer for one-point increment in the overall World Cancer Research Fund/American Institute for Cancer Research (WCRF/AICR) score excluding alternately each recommendation at a time, Italy, 1992–2006.

Table 3 shows the OR of endometrial cancer according to the overall WCRF/AICR score and the dietary WCRF/AICR score. After allowing for major confounders, high adherence to the WCRF/AICR recommendations was inversely related to the risk of endometrial cancer, with an OR of 0·42 (95 % CI 0·30, 0·61) for the highest compared with the lowest score quartile (*P*-value for trend < 0·001). The OR for one-point increment in the WCRF/AICR score was 0·72 (95 % CI 0·63, 0·83). As for the dietary WCRF/AICR score, the OR for the highest compared with the lowest tertile was 0·67 (95 % CI 0·46, 0·96, *P*-value for trend = 0·017), and that for one-point increment was 0·81 (95 % CI 0·68, 0·96). Results were virtually identical when using a complete case approach (OR = 0·42, 95 % CI 0·29, 0·60 for the highest compared with the lowest overall WCRF/AICR score quartile; OR = 0·66, 95 % CI 0·47, 0·94 for the highest compared with the lowest dietary WCRF/AICR score tertile). The inverse association with the overall WCRF/AICR score was consistent after the exclusion alternately of each recommendation on diet and physical activity at a time (Fig. 1). When the recommendation on body fatness was excluded, the association was reduced (OR for one-point increment = 0·90, 95 % CI 0·77, 1·05).

**Table 3.** Association of the overall World Cancer Research Fund/American Institute for Cancer Research (WCRF/AICR) score and the WCRF/AICR diet score with endometrial cancer risk, Italy, 1992–2006 (Odds ratios and 95 % confidence intervals; numbers and percentages)

	Cases, *n*	%	Controls, *n*	%	OR*	95 % CI
WCRF/AICR score, quartiles						
I (< 3·25)	147	32·4	191	21·0	1†	
II (3·25–3·99)	158	34·8	307	33·8	0·70	0·51, 0·96
III (4·00–4·49)	70	15·4	170	18·7	0·57	0·39, 0·82
IV (≥ 4·50)	79	17·4	240	26·4	0·42	0·30, 0·61
*P*-value for trend					< 0·001	
One-point increment					0·72	0·63, 0·83
WCRF/AICR diet score, tertiles						
I (< 2·25)	122	26·9	226	24·9	1†	
II (2·25–2·99)	210	46·3	357	39·3	1·05	0·77, 1·42
III (≥ 3·00)	122	26·9	325	35·8	0·67	0·46, 0·94
*P*-value for trend					0·017	
One-point increment					0·81	0·68, 0·96

* Estimated from logistic regression models conditioned on age and centre and including terms for year of interview, education, smoking, total energy intake, history of diabetes, age at menarche, menopausal status, parity, use of oral contraceptives and hormone replacement therapy. OR according to the WCRF/AIRC diet score were further adjusted for BMI and physical activity.

† Reference category.

In subgroup analyses (Table 4), the association was stronger among women with a normal weight, those who were older, and consequently those in post-menopause and those with ≥ 2 children.

**Table 4.** Association between the overall World Cancer Research Fund/American Institute for Cancer Research (WCRF/AICR) score and endometrial cancer risk in strata of selected covariates, Italy, 1992–2006 (Odds ratios and 95 % confidence intervals)

	Cases:controls	OR* for One-point increment	95 % CI	*P*-interaction
Strata				
Age				
< 55	126:252	0·89	0·69, 1·15	
55–69	247:494	0·65	0·54, 0·78	
≥ 70	81:162	0·65	0·45, 0·94	0·024
BMI (kg/m^2^)†				
< 25	126:413	0·80	0·62, 1·04	
25–29·9	160:351	0·93	0·73, 1·20	
≥ 30	168:140	1·13	0·80, 1·59	0·061
Menopausal status				
Pre/Peri	83:174	0·90	0·66, 1·25	
Post	360:726	0·69	0·59, 0·81	0·091
Parity				
0–1	160:276	0·82	0·65, 1·02	
≥ 2	294:632	0·68	0·58, 0·82	0·093
Hormone replacement therapy use				
Never	405:830	0·72	0·63, 0·83	
Ever	49:78	0·77	0·42, 1·42	0·445

* Estimated from logistic regression models conditioned on age and centre and including terms for year of interview, education, smoking, total energy intake, history of diabetes, age at menarche, menopausal status, parity, use of oral contraceptives and hormone replacement therapy, unless the variable was the stratification factor.

† OR of endometrial cancer for the WCRF/AICR score excluding BMI: (1) < 25: 0·78 (95 % CI 0·59, 1·02), (2) 25–29: 0·92 (95 % CI 0·72, 1·19) and (3) ≥ 30: 1·13 (95 % CI 0·80, 1·59).

## Discussion

In this large, multicentric Italian study, greater adherence to the WCRF/AICR preventive cancer recommendations on body fatness, physical activity, and diet was associated with an approximately 60 % reduced risk of endometrial cancer. As expected^(52)^, body weight had the strongest influence on the risk; however, a score measuring adherence to the recommendations related to diet was inversely associated with the risk of endometrial cancer after adjusting for BMI. In addition, the inverse relation was stronger in normal weight women, reflecting the key role of overweight and obesity on endometrial cancer risk^(52)^.

Maintaining a healthy weight throughout life – of specific importance for endometrial cancer risk – being physically active, following a healthy eating pattern and avoiding alcohol use are the key recommendations for the prevention of cancer, also according to the American Cancer Society^(53,54)^.

Our results on body fatness reflect the well-established and strong association between overweight, obesity and endometrial cancer risk. Obesity (defined as BMI > 30 or < 35 kg/m^2^) is associated with an over 2-fold increase in the risk of endometrial cancer and severe obesity (defined as BMI > 35 kg/m^2^) with a 5-fold increase^(55)^. The relationship involves the hyper-oestrogenic state of obesity^(56)^, besides other mechanisms. Adipose tissue, functioning as an important endocrine organ, contributes to hormone production (such as oestrogens), maintenance of a pro-inflammatory state, and stimulation of cellular proliferation pathways. Such factors play a key role in carcinogenesis and endometrial proliferation. In addition, adiposity influences the metabolism and is associated with insulin resistance and hyperinsulinaemia, well-recognised risk factors for the endometrial cancer^(57)^. Intentional weight loss (self-reported or after bariatric surgery) and maintaining a stable weight were related to a significantly lower risk of endometrial cancer (relative risk ranging from 0·61 to 0·96)^(58)^. A study conducted in a cohort of severely obese women undergoing a weight loss intervention including diet and physical activity found that levels of cancer-associated biomarkers could be normalised with weight loss^(59)^.

As for physical activity, the WHO^(60)^ and the US Physical Activity Guidelines Advisory committee^(61)^, on the basis of their appraisal of a number of systematic reviews and meta-analyses, reported a moderate to high-certainty evidence that high physical activity levels are associated with a reduction in endometrial cancer risk. A systematic review and meta-analysis^(62)^ reported a significant inverse association between physical activity and endometrial cancer among overweight or obese women only, possibly due to the counterbalance function of physical activity against the unfavourable effects of obesity and the different composition of body mass. Further, since physical activity and BMI are strongly linked, when the benefit from physical activity in preventing endometrial cancer has been explored using a mediation analysis, it appeared that the majority of the protective role was mediated through a reduction in the risk of obesity^(63)^. Other mechanisms involved may be the decreasing oestrogens through reducing peripheral adipose tissue where the conversion of androgens to oestrogens occurs^(64)^, the improvement of insulin sensitivity^(65)^, the alteration of the insulin-like growth factor axis^(66)^, and the reduction of pro-inflammatory mediators^(67)^. We measured adherence to the recommendation on physical activity combining available questionnaire data on the level of physical activity at work and on the time spent in leisure time physical activity at age 30–39 years and adapted cut points for adherence proposed by the standard scoring system, which were expressed as min/week of moderate-vigorous physical activity, to our physical activity variable. With such an approach, less than 10 % of cases and controls were categorised as ‘non-adherent’, and we did not find any relevant association with endometrial cancer. Whether higher levels of physical activity may favourably affect endometrial cancer risk cannot be excluded.

As for the WCRF/AICR recommendations on diet, various studies showed a favourable role of dietary fibre^(18,19)^, fruit^(20)^, and vegetables^(20–22)^ on endometrial cancer risk. Vegetables and fruit represent a source of a variety of micronutrients and other bioactive constituents that may protect from cancer through modulation of steroid hormone concentration and metabolism, antioxidant activities, modulation of detoxification enzymes, and stimulation of the immune system^(68)^. As for dietary fibres, the favourable role may be attributable to the decrease in plasma cholesterol levels and in postprandial glycaemia, and the bacterial fermentation of fibre to short-chain fatty acids^(69)^. Conversely, the intake of red and processed meat was directly associated with endometrial cancer risk in some^(12–14)^, but not all studies^(21,70)^; alcohol intake was not appreciably associated with the disease^(71,72)^ and the few studies investigating sugar-sweetened beverage consumption^(14,73,74)^ gave inconsistent results. In our study, a score reflecting adherence to a dietary pattern characterised by high consumption of vegetables, fruit and wholegrains and low consumption of energy-dense food, red and processed meat and sugar-sweetened and alcoholic drinks reduced the risk of endometrial cancer. Along this line, previous studies found inverse associations with healthy eating behaviours, including the Mediterranean diet^(75–77)^ and, more recently, a diet for diabetes prevention^(78)^, and direct associations with Western-style dietary patterns^(79,80)^.

We followed the standardised scoring system for the operationalization of the WCRF/AICR recommendations developed by a collaborative group including, among the others, researchers from the US National Cancer Institute and WCRF/AICR Continuous Update Project Expert Panel in order to improve comparability and consistency across studies^(50,51)^. We were unable to include information on waist circumference in the body fatness recommendation because the self-reported waist circumference measure was not available for 147 cases and 314 controls; we adapted the recommendation on physical activity according to data availability; we used energy density as a *proxy* for the consumption of *fast foods* and other processed foods high in fat, starches or sugars, whose consumption was not specifically collected by the FFQ and we did not consider the optional recommendation on breastfeeding.

Selection bias should be limited in our study, as we excluded from the control group women admitted to hospitals for hormone-related or gynaecologic conditions or any disease leading to long-term modifications in diet. Moreover, a low refusal rate was observed and the recruitment areas were similar for cases and controls. With reference to information bias, it was limited through the direct interview of cases and controls by the same trained interviewers in similar hospital conditions. In addition, we analysed the impact of the adherence to the WCRF/AICR score proposed in 2018 on data collected between 1992 and 2006 in a population unaware of those recommendations. Weight and height were self-reported, and BMI tended, therefore, to be underestimated, but this is unlikely to be differential between cases and controls. Finally, among limitations, information on grade, stage and possible therapy of cancer cases was not available; however, these factors are unlikely to materially influence diet-related associations. The relatively large sample size, the satisfactory reproducibility^(46,47)^ and validity^(48)^ of the FFQ and the allowance for several potential confounding factors represented the strengths of the study.

In conclusion, in this study higher adherence to the WCRF/AICR recommendations was associated with about 60 % reduced risk of endometrial cancer; while body weight had the strongest influence on the risk, a score considering only recommendations related to diet decreased the risk as well. Maintaining a healthy weight throughout life is the key recommendation for the prevention of this neoplasm. Being physically active and follow a healthy diet may also contribute to endometrial cancer prevention.

## Supplementary material

For supplementary material accompanying this paper visit https://doi.org/10.1017/S0007114522002872. click here to view supplementary material

## References

[1] Sung H , Ferlay J , Siegel RL , et al. (2021) Global Cancer Statistics 2020: GLOBOCAN estimates of incidence and mortality worldwide for 36 cancers in 185 countries. CA Cancer J Clin 71, 209–249. 33538338 10.3322/caac.21660

[2] Lortet-Tieulent J , Ferlay J , Bray F , et al. (2018) International patterns and trends in endometrial cancer incidence, 1978–2013. J Natl Cancer Inst 110, 354–361. 29045681 10.1093/jnci/djx214

[3] Tempfer CB , Hilal Z , Kern P , et al. (2020) Menopausal hormone therapy and risk of endometrial cancer: a systematic review. Cancers 12, 2195. 32781573 10.3390/cancers12082195 PMC7465414

[4] Hopkins BD , Goncalves MD & Cantley LC (2016) Obesity and cancer mechanisms: cancer metabolism. J Clin Oncol 34, 4277–4283. 27903152 10.1200/JCO.2016.67.9712 PMC5562429

[5] Kerr J , Anderson C & Lippman SM (2017) Physical activity, sedentary behaviour, diet, and cancer: an update and emerging new evidence. Lancet Oncol 18, e457–e471. 28759385 10.1016/S1470-2045(17)30411-4 PMC10441558

[6] Lauby-Secretan B , Scoccianti C , Loomis D , et al. (2016) Body fatness and cancer--viewpoint of the IARC working group. N Engl J Med 375, 794–798. 27557308 10.1056/NEJMsr1606602 PMC6754861

[7] Friedenreich C , Cust A , Lahmann PH , et al. (2007) Physical activity and risk of endometrial cancer: the European prospective investigation into cancer and nutrition. Int J Cancer 121, 347–355. 17357139 10.1002/ijc.22676

[8] Shen D , Mao W , Liu T , et al. (2014) Sedentary behavior and incident cancer: a meta-analysis of prospective studies. PLoS One 9, e105709. 25153314 10.1371/journal.pone.0105709 PMC4143275

[9] Lacey JV Jr , Chia VM , Rush BB , et al. (2012) Incidence rates of endometrial hyperplasia, endometrial cancer and hysterectomy from 1980 to 2003 within a large prepaid health plan. Int J Cancer 131, 1921–1929. 22290745 10.1002/ijc.27457

[10] Hatami Marbini M , Amiri F & Sajadi Hezaveh Z (2021) Dietary glycemic index, glycemic load, insulin index, insulin load and risk of diabetes-related cancers: a systematic review of cohort studies. Clin Nutr ESPEN 42, 22–31. 33745582 10.1016/j.clnesp.2021.02.008

[11] Turati F , Galeone C , Augustin LSA , et al. (2019) Glycemic index, glycemic load and cancer risk: an updated meta-analysis. Nutrients 11, 2342. 31581675 10.3390/nu11102342 PMC6835610

[12] Rosato V , Negri E , Parazzini F , et al. (2018) Processed meat and selected hormone-related cancers. Nutrition 49, 17–23. 29571606 10.1016/j.nut.2017.10.025

[13] Farvid MS , Sidahmed E , Spence ND , et al. (2021) Consumption of red meat and processed meat and cancer incidence: a systematic review and meta-analysis of prospective studies. Eur J Epidemiol 36, 937–951. 34455534 10.1007/s10654-021-00741-9

[14] Dunneram Y , Greenwood DC & Cade JE (2019) Diet and risk of breast, endometrial and ovarian cancer: UK women’s cohort study. Br J Nutr 122, 564–574. 30526696 10.1017/S0007114518003665 PMC6763332

[15] Lukic M , Guha N , Licaj I , et al. (2018) Coffee drinking and the risk of endometrial cancer: an updated meta-analysis of observational studies. Nutr Cancer 70, 513–528. 29708405 10.1080/01635581.2018.1460681

[16] Di Maso M , Boffetta P , Negri E , et al. (2021) Caffeinated coffee consumption and health outcomes in the US population: a dose-response meta-analysis and estimation of disease cases and deaths avoided. Adv Nutr 12, 1160–1176. 33570108 10.1093/advances/nmaa177 PMC8321867

[17] Bravi F , Scotti L , Bosetti C , et al. (2009) Coffee drinking and endometrial cancer risk: a metaanalysis of observational studies. Am J Obstetrics Gynecology 200, 130–135. 10.1016/j.ajog.2008.10.032 19110217

[18] Chen K , Zhao Q , Li X , et al. (2018) Dietary fiber intake and endometrial cancer risk: a systematic review and meta-analysis. Nutrients 10, 945. 30037138 10.3390/nu10070945 PMC6073518

[19] Li H , Mao H , Yu Y , et al. (2020) Association between dietary fiber and endometrial cancer: a meta-analysis. Nutr Cancer 72, 959–967. 31584301 10.1080/01635581.2019.1670218

[20] Bandera EV , Kushi LH , Moore DF , et al. (2007) Fruits and vegetables and endometrial cancer risk: a systematic literature review and meta-analysis. Nutr Cancer 58, 6–21. 17571962 10.1080/01635580701307929

[21] Bravi F , Scotti L , Bosetti C , et al. (2009) Food groups and endometrial cancer risk: a case-control study from Italy. Am J Obstet Gynecol 200, 293.e291–297. 10.1016/j.ajog.2008.09.015 19091304

[22] Turati F , Rossi M , Pelucchi C , et al. (2015) Fruit and vegetables and cancer risk: a review of southern European studies. Br J Nutr 113, S102–110. 26148912 10.1017/S0007114515000148

[23] Biel RK , Csizmadi I , Cook LS , et al. (2011) Risk of endometrial cancer in relation to individual nutrients from diet and supplements. Public Health Nutr 14, 1948–1960. 21752313 10.1017/S1368980011001066

[24] Wiseman M (2008) The second World Cancer Research Fund/American Institute for Cancer Research expert report. food, nutrition, physical activity, and the prevention of cancer: a global perspective. Proc Nutr Soc 67, 253–256. 18452640 10.1017/S002966510800712X

[25] Clinton SK , Giovannucci EL & Hursting SD (2020) The World Cancer Research Fund/American Institute for Cancer Research third expert report on diet, nutrition, physical activity, and cancer: impact and future directions. J Nutr 150, 663–671. 31758189 10.1093/jn/nxz268 PMC7317613

[26] Vergnaud AC , Romaguera D , Peeters PH , et al. (2013) Adherence to the World Cancer Research Fund/American Institute for Cancer Research guidelines and risk of death in Europe: results from the European Prospective Investigation into Nutrition and Cancer cohort study. Am J Clin Nutr 97, 1107–1120. 23553166 10.3945/ajcn.112.049569

[27] Kaluza J , Harris HR , Hakansson N , et al. (2020) Adherence to the WCRF/AICR 2018 recommendations for cancer prevention and risk of cancer: prospective cohort studies of men and women. Br J Cancer 122, 1562–1570. 32210367 10.1038/s41416-020-0806-x PMC7217975

[28] Korn AR , Reedy J , Brockton NT , et al. (2022) The 2018 World Cancer Research Fund/American Institute for Cancer Research score and cancer risk: a longitudinal analysis in the NIH-AARP diet and health study. Cancer Epidemiol Biomarkers Prev (In Press). 10.1158/1055-9965.EPI-22-0044 PMC9532348 35877953

[29] Turati F , Dalmartello M , Bravi F , et al. (2020) Adherence to the World Cancer Research Fund/American Institute for Cancer Research recommendations and the risk of breast cancer. Nutrients 12, 607. 32110887 10.3390/nu12030607 PMC7146587

[30] Karavasiloglou N , Husing A , Masala G , et al. (2019) Adherence to the World Cancer Research Fund/American Institute for Cancer Research cancer prevention recommendations and risk of *in situ* breast cancer in the European Prospective Investigation into Cancer and Nutrition (EPIC) cohort. BMC Med 17, 221. 31787099 10.1186/s12916-019-1444-0 PMC6886197

[31] Kaledkiewicz E & Szostak-Wegierek D (2019) Current and past adherence to the World Cancer Research Fund/American Institute for Cancer Research recommendations in survivors of breast cancer. Rocz Panstw Zakl Hig 70, 295–305. 31515989 10.32394/rpzh.2019.0081

[32] Barrios-Rodriguez R , Toledo E , Martinez-Gonzalez MA , et al. (2020) Adherence to the 2018 World Cancer Research Fund/American Institute for Cancer Research recommendations and breast cancer in the SUN project. Nutrients 12, 2076. 32668662 10.3390/nu12072076 PMC7400833

[33] Petimar J , Smith-Warner SA , Rosner B , et al. (2019) Adherence to the World Cancer Research Fund/American Institute for Cancer Research 2018 recommendations for cancer prevention and risk of colorectal cancer. Cancer Epidemiol Biomarkers Prev 28, 1469–1479. 31235471 10.1158/1055-9965.EPI-19-0165 PMC6726499

[34] Onyeaghala G , Lintelmann AK , Joshu CE , et al. (2020) Adherence to the World Cancer Research Fund/American Institute for Cancer Research cancer prevention guidelines and colorectal cancer incidence among African Americans and whites: the Atherosclerosis Risk in Communities study. Cancer 126, 1041–1050. 31873947 10.1002/cncr.32616 PMC7021569

[35] van Veen MR , Mols F , Bours MJL , et al. (2019) Adherence to the World Cancer Research Fund/American Institute for Cancer Research recommendations for cancer prevention is associated with better health-related quality of life among long-term colorectal cancer survivors: results of the PROFILES registry. Support Care Cancer 27, 4565–4574. 30927111 10.1007/s00520-019-04735-y PMC6825038

[36] El Kinany K , Huybrechts I , Kampman E , et al. (2019) Concordance with the World Cancer Research Fund/American Institute for Cancer Research recommendations for cancer prevention and colorectal cancer risk in morocco: a large, population-based case-control study. Int J Cancer 145, 1829–1837. 30861106 10.1002/ijc.32263

[37] Winkels RM , van Lee L , Beijer S , et al. (2016) Adherence to the World Cancer Research Fund/American Institute for Cancer Research lifestyle recommendations in colorectal cancer survivors: results of the PROFILES registry. Cancer Med 5, 2587–2595. 27418442 10.1002/cam4.791 PMC5055169

[38] Turati F , Bravi F , Di Maso M , et al. (2017) Adherence to the World Cancer Research Fund/American Institute for Cancer Research recommendations and colorectal cancer risk. Eur J Cancer 85, 86–94. 28892777 10.1016/j.ejca.2017.08.015

[39] Kenkhuis MF , van der Linden BWA , Breedveld-Peters JJL , et al. (2021) Associations of the dietary World Cancer Research Fund/American Institute for Cancer Research (WCRF/AICR) recommendations with patient-reported outcomes in colorectal cancer survivors 2–10 years post-diagnosis: a cross-sectional analysis. Br J Nutr 125, 1188–1200. 33087189 10.1017/S0007114520003487

[40] Zhang ZQ , Li QJ , Hao FB , et al. (2020) Adherence to the 2018 World Cancer Research Fund/American Institute for Cancer Research cancer prevention recommendations and pancreatic cancer incidence and mortality: a prospective cohort study. Cancer Med 9, 6843–6853. 32716590 10.1002/cam4.3348 PMC7520356

[41] Lucas AL , Bravi F , Boffetta P , et al. (2016) Adherence to World Cancer Research Fund/American Institute for Cancer Research recommendations and pancreatic cancer risk. Cancer Epidemiol 40, 15–21. 26605429 10.1016/j.canep.2015.10.026 PMC5494828

[42] Olmedo-Requena R , Lozano-Lorca M , Salcedo-Bellido I , et al. (2020) Compliance with the 2018 World Cancer Research Fund/American Institute for Cancer Research cancer prevention recommendations and prostate cancer. Nutrients 12, 768. 32183345 10.3390/nu12030768 PMC7146507

[43] Bravi F , Polesel J , Garavello W , et al. (2017) Adherence to the World Cancer Research Fund/American Institute for Cancer Research recommendations and head and neck cancers risk. Oral Oncol 64, 59–64. 28024725 10.1016/j.oraloncology.2016.11.012

[44] Bravi F , Bertuccio P , Turati F , et al. (2015) Nutrient-based dietary patterns and endometrial cancer risk: an Italian case-control study. Cancer Epidemiol 39, 66–72. 25556109 10.1016/j.canep.2014.12.003

[45] Rossi M , Edefonti V , Parpinel M , et al. (2013) Proanthocyanidins and other flavonoids in relation to endometrial cancer risk: a case-control study in Italy. Br J Cancer 109, 1914–1920. 23922105 10.1038/bjc.2013.447 PMC3790154

[46] Franceschi S , Barbone F , Negri E , et al. (1995) Reproducibility of an Italian food frequency questionnaire for cancer studies. Results for specific nutrients. Ann Epidemiol 5, 69–75. 7728288 10.1016/1047-2797(95)92893-d

[47] Franceschi S , Negri E , Salvini S , et al. (1993) Reproducibility of an Italian food frequency questionnaire for cancer studies: results for specific food items. Eur J Cancer 29A, 2298–2305. 8110502 10.1016/0959-8049(93)90225-5

[48] Decarli A , Franceschi S , Ferraroni M , et al. (1996) Validation of a food-frequency questionnaire to assess dietary intakes in cancer studies in Italy. Results for specific nutrients. Ann Epidemiol 6, 110–118. 8775590 10.1016/1047-2797(95)00129-8

[49] Gnagnarella P , Parpinel M & Salvini S (2004) The update of the Italian food composition database. J Food Comp Anal 17, 509–522.

[50] Shams-White MM , Brockton NT , Mitrou P , et al. (2019) Operationalizing the 2018 World Cancer Research Fund/American Institute for Cancer Research (WCRF/AICR) cancer prevention recommendations: a standardized scoring system. Nutrients 11, 1572. 31336836 10.3390/nu11071572 PMC6682977

[51] Shams-White MM , Romaguera D , Mitrou P , et al. (2020) Further guidance in implementing the standardized 2018 World Cancer Research Fund/American Institute for Cancer Research (WCRF/AICR) score. Cancer Epidemiol Biomarkers Prev 29, 889–894. 32152215 10.1158/1055-9965.EPI-19-1444

[52] Parazzini F , La Vecchia C , Bocciolone L , et al. (1991) The epidemiology of endometrial cancer. Gynecol Oncol 41, 1–16. 2026352 10.1016/0090-8258(91)90246-2

[53] Kushi LH , Doyle C , McCullough M , et al. (2012) American Cancer Society Guidelines on nutrition and physical activity for cancer prevention: reducing the risk of cancer with healthy food choices and physical activity. CA Cancer J Clin 62, 30–67. 22237782 10.3322/caac.20140

[54] Rock CL , Thomson C , Gansler T , et al. (2020) American Cancer Society guideline for diet and physical activity for cancer prevention. CA Cancer J Clin 70, 245–271. 32515498 10.3322/caac.21591

[55] Shaw E , Farris M , McNeil J , et al. (2016) Obesity and endometrial cancer. Recent Results Cancer Res 208, 107–136. 27909905 10.1007/978-3-319-42542-9_7

[56] Kitson S & Crosbie E (2019) Endometrial cancer and obesity. Obstet Gynecol 21, 237–245.

[57] Orgel E & Mittelman SD (2013) The links between insulin resistance, diabetes, and cancer. Curr Diab Rep 13, 213–222. 23271574 10.1007/s11892-012-0356-6 PMC3595327

[58] Zhang X , Rhoades J , Caan BJ , et al. (2019) Intentional weight loss, weight cycling, and endometrial cancer risk: a systematic review and meta-analysis. Int J Gynecol Cancer 29, 1361–1371. 31451560 10.1136/ijgc-2019-000728 PMC6832748

[59] Linkov F , Maxwell GL , Felix AS , et al. (2012) Longitudinal evaluation of cancer-associated biomarkers before and after weight loss in RENEW study participants: implications for cancer risk reduction. Gynecol Oncol 125, 114–119. 22198242 10.1016/j.ygyno.2011.12.439 PMC4387771

[60] Bull FC , Al-Ansari SS , Biddle S , et al. (2020) World Health Organization 2020 guidelines on physical activity and sedentary behaviour. Br J Sports Med 54, 1451–1462. 33239350 10.1136/bjsports-2020-102955 PMC7719906

[61] Committee PAGA (2018) 2018 Physical Activity Guidelines Advisory Committee Scientific Report. Washington, DC: U.S. Department of Health and Human Services. F4–18.

[62] Schmid D , Behrens G , Keimling M , et al. (2015) A systematic review and meta-analysis of physical activity and endometrial cancer risk. Eur J Epidemiol 30, 397–412. 25800123 10.1007/s10654-015-0017-6

[63] Saint-Maurice PF , Sampson JN , Michels KA , et al. (2021) Physical activity from adolescence through midlife and associations with body mass index and endometrial cancer risk. JNCI Cancer Spectr 5, pkab065. 34476340 10.1093/jncics/pkab065 PMC8406434

[64] Kaaks R , Lukanova A & Kurzer MS (2002) Obesity, endogenous hormones, and endometrial cancer risk: a synthetic review. Cancer Epidemiol Biomarkers Prev 11, 1531–1543. 12496040

[65] Mann S , Beedie C , Balducci S , et al. (2014) Changes in insulin sensitivity in response to different modalities of exercise: a review of the evidence. Diabetes Metab Res Rev 30, 257–268. 24130081 10.1002/dmrr.2488

[66] Gatti R , De Palo EF , Antonelli G , et al. (2012) IGF-I/IGFBP system: metabolism outline and physical exercise. J Endocrinol Invest 35, 699–707. 22714057 10.3275/8456

[67] Bruunsgaard H (2005) Physical activity and modulation of systemic low-level inflammation. J Leukoc Biol 78, 819–835. 16033812 10.1189/jlb.0505247

[68] Lampe JW (1999) Health effects of vegetables and fruit: assessing mechanisms of action in human experimental studies. Am J Clin Nutr 70, 475S–490S. 10479220 10.1093/ajcn/70.3.475s

[69] Slavin J (2013) Fiber and prebiotics: mechanisms and health benefits. Nutrients 5, 1417–1435. 23609775 10.3390/nu5041417 PMC3705355

[70] Cross AJ , Leitzmann MF , Gail MH , et al. (2007) A prospective study of red and processed meat intake in relation to cancer risk. PLoS Med 4, e325. 18076279 10.1371/journal.pmed.0040325 PMC2121107

[71] Fedirko V , Jenab M , Rinaldi S , et al. (2013) Alcohol drinking and endometrial cancer risk in the European Prospective Investigation into Cancer and Nutrition (EPIC) study. Ann Epidemiol 23, 93–98. 23273691 10.1016/j.annepidem.2012.11.009

[72] Turati F , Gallus S , Tavani A , et al. (2010) Alcohol and endometrial cancer risk: a case-control study and a meta-analysis. Cancer Causes Control 21, 1285–1296. 20396942 10.1007/s10552-010-9556-z

[73] Arthur RS , Kirsh VA , Mossavar-Rahmani Y , et al. (2021) Sugar-containing beverages and their association with risk of breast, endometrial, ovarian and colorectal cancers among Canadian women. Cancer Epidemiol 70, 101855. 33220638 10.1016/j.canep.2020.101855

[74] Inoue-Choi M , Robien K , Mariani A , et al. (2013) Sugar-sweetened beverage intake and the risk of type I and type II endometrial cancer among postmenopausal women. Cancer Epidemiol Biomarkers Prev 22, 2384–2394. 24273064 10.1158/1055-9965.EPI-13-0636 PMC3892378

[75] Filomeno M , Bosetti C , Bidoli E , et al. (2015) Mediterranean diet and risk of endometrial cancer: a pooled analysis of three Italian case-control studies. Br J Cancer 112, 1816–1821. 26010500 10.1038/bjc.2015.153 PMC4647248

[76] Ricceri F , Giraudo MT , Fasanelli F , et al. (2017) Diet and endometrial cancer: a focus on the role of fruit and vegetable intake, Mediterranean diet and dietary inflammatory index in the endometrial cancer risk. BMC Cancer 17, 757. 29132343 10.1186/s12885-017-3754-y PMC5683600

[77] Zhang YH , Li Z & Tan MZ (2021) Association between diet quality and risk of ovarian and endometrial cancers: a systematic review of epidemiological studies. Front Oncol 11, 659183. 34084748 10.3389/fonc.2021.659183 PMC8168438

[78] Esposito G , Bravi F , Serraino D , et al. (2021) Diabetes risk reduction diet and endometrial cancer risk. Nutrients 13, 2630. 34444790 10.3390/nu13082630 PMC8399314

[79] Si CJ , Shu L , Zheng PF , et al. (2017) Dietary patterns and endometrial cancer: a meta-analysis. Eur J Cancer Prev 26, 336–345. 27139775 10.1097/CEJ.0000000000000266

[80] Alizadeh S , Djafarian K , Alizadeh M , et al. (2020) The relation of healthy and Western dietary patterns to the risk of endometrial and ovarian cancers: a systematic review and meta-analysis. Int J Vitam Nutr Res 90, 365–375. 30806608 10.1024/0300-9831/a000514

