# Supplementary material for: Adherence to the World Cancer Research Fund/American Institute for Cancer Research recommendations and endometrial cancer risk: a multicentric case–control study
Source: Br J Nutr. 2022 Sep 12;129(12):2133–41. doi: 10.1017/S0007114522002872 (PMC10197087; doi:10.1017/S0007114522002872)
Supplement: Supplementary file 1 [file S0007114522002872sup.zip › S0007114522002872sup001.docx]

**Table S1.** Operationalization of the 2018 WCRF/AICR recommendations.

| *2018 WCRF/AICR recommendations* ^a^ | *2018 WCRF/AICR score ^1,2^* | *Points* | *Operationalization/Comments* | *Points* |
| --- | --- | --- | --- | --- |
| 1. Be at a healthy weight | BMI (kg/m^2^) |  | BMI (kg/m^2^) |  |
|  | 18.5 - 24.9 | 0.5 | 18.5 - 24.9 | 1 |
|  | 25 - 29.9 | 0.25 | 25 - 29.9 | 0.5 |
|  | <18.5 or ≥30 | 0 | <18.5 or ≥30 | 0 |
|  | Waist circumference (cm) |  | Waist circumference (cm) |  |
|  | Men: <94 Women: <80 | 0.5 | Insufficient data ^b^ | - |
|  | Men: 94-<102 Women:80-<88 | 0.25 |  |  |
|  | Men: ≥102 Women: ≥88 | 0 |  |  |
| 2. Be physically active | Total moderate-vigorous physical activity  (min/week) | | Total moderate-vigorous physical activity (at age 30-39 years,  both occupational and leisure time physical activity) | |
|  | ≥150 | 1 | Very heavy/heavy job or ≥ 5 hours/week of leisure time physical activity | 1 |
|  | 75-<150 | 0.5 | Medium job and ≤ 4 hours/week of leisure time physical activity or standing/sedentary job and 2-4 hours/week of leisure time physical activity | 0.5 |
|  | <75 | 0 | Sedentary job and <2 hours/week of leisure time physical activity | 0 |
| 3. Eat a diet rich in wholegrains, vegetables and fruit | Fruits and vegetables (g/die) |  | Fruits and vegetables (g/die) |  |
|  | ≥400 | 0.5 | ≥400 | 0.5 |
|  | 200-<400 | 0.25 | 200-<400 | 0.25 |
|  | <200 | 0 | <200 | 0 |
|  | Total fiber (g/die): |  | Total fiber (g/die): |  |
|  | ≥30 | 0.5 | ≥30 | 0.5 |
|  | 15-<30 | 0.25 | 15-<30 | 0.25 |
|  | <15 | 0 | <15 | 0 |
| 4. Limit consumption of *fast foods* and other processed foods high in fat, starches or sugars | Percent of total kcal from ultra-processed foods | | Energy dense foods (kcal/100 g/die) |  |
|  | Tertile 1 | 1 | Energy density: ≤125 | 1 |
|  | Tertile 2 | 0.5 | Energy density: 125-<175 | 0.5 |
|  | Tertile 3 | 0 | Energy density: ≥175 | 0 |
| 5. Limit consumption of red and processed meat | Total red meat (g/week) and processed meat (g/week) | | Total red meat (g/week) and processed meat (g/week) | |
|  | Red meat <500 and processed meat <21 | 1 | Red and processed meat <500 and processed meat <21 | 1 |
|  | Red meat <500 and processed meat 21-<100 | 0.5 | Red and processed meat <500 and processed meat 21-<100 | 0.5 |
|  | Red meat >500 or processed meat ≥100 | 0 | Red and processed meat ≥500 or processed meat ≥100 | 0 |
| 6. Limit consumption of sugar sweetened drinks | Total sugar-sweetened drinks (g/die) |  | Total sugar-sweetened drinks (g/die) |  |
|  | 0 |  | 0 | 1 |
|  | 0-≤250 |  | 0-≤250 | 0.5 |
|  | >250 |  | >250 | 0 |
| 7) Avoid alcohol consumption | Total ethanol (g/die): |  | Alcoholic drinks (n/week) |  |
|  | 0 | 1 | 0 | 1 |
|  | Women: >0-≤14 (1 drink) [Men: >0-≤28 (2 drinks)] | 0.5 | >0-≤7 | 0.5 |
|  | Women: >14 (1 drink) [Men: >28 (2 drinks)] | 0 | >7 | 0 |

^a^ The optional recommendation on breastfeeding was not included ^b^ Data on waist circumference were not available for 147 cases and 314 controls. AICR: American Institute for Cancer Research; BMI: body mass index; WCRF: World Cancer Research Fund.

1 Shams-White, M. M. *et al.* Operationalizing the 2018 World Cancer Research Fund/American Institute for Cancer Research (WCRF/AICR) Cancer Prevention Recommendations: A Standardized Scoring System. *Nutrients* **11** (2019).

2 Shams-White, M. M. *et al.* Further Guidance in Implementing the Standardized 2018 World Cancer Research Fund/American Institute for Cancer Research (WCRF/AICR) Score. *Cancer Epidemiol Biomarkers Prev* **29**, 889-894 (2020).
